# Highly colloidally stable trimodal ^125^I-radiolabeled PEG-neridronate-coated upconversion/magnetic bioimaging nanoprobes

**DOI:** 10.1038/s41598-020-77112-z

**Published:** 2020-11-18

**Authors:** Uliana Kostiv, Jan Kučka, Volodymyr Lobaz, Nikolay Kotov, Olga Janoušková, Miroslav Šlouf, Bartosz Krajnik, Artur Podhorodecki, Pavla Francová, Luděk Šefc, Daniel Jirák, Daniel Horák

**Affiliations:** 1 Institute of Macromolecular Chemistry, Czech Academy of Sciences, Heyrovského nám. 2, 162 06 Prague 6, Czech Republic; 2 Department of Experimental Physics, Wroclaw University of Science and Technology, Wybrzeze Wyspianskiego 27, 50-370 Wroclaw, Poland; 3 Center for Advanced Preclinical Imaging (CAPI), First Faculty of Medicine, Charles University, Salmovská 3, 120 00 Prague 2, Czech Republic; 4 Department of Diagnostic and Interventional Radiology, Institute for Clinical and Experimental Medicine, Vídeňská 1958/9, 140 21 Prague 4, Czech Republic; 5 Institute of Biophysics and Informatics, First Faculty of Medicine, Charles University, Salmovská 1, 120 00 Prague 2, Czech Republic

**Keywords:** Chemistry, Materials science, Nanoscience and technology

## Abstract

“All-in-one” multifunctional nanomaterials, which can be visualized simultaneously by several imaging techniques, are required for the efficient diagnosis and treatment of many serious diseases. This report addresses the design and synthesis of upconversion magnetic NaGdF_4_:Yb^3+^/Er^3+^(Tm^3+^) nanoparticles by an oleic acid-stabilized high-temperature coprecipitation of lanthanide precursors in octadec-1-ene. The nanoparticles, which emit visible or UV light under near-infrared (NIR) irradiation, were modified by in-house synthesized PEG-neridronate to facilitate their dispersibility and colloidal stability in water and bioanalytically relevant phosphate buffered saline (PBS). The cytotoxicity of the nanoparticles was determined using HeLa cells and human fibroblasts (HF). Subsequently, the particles were modified by Bolton-Hunter-neridronate and radiolabeled by ^125^I to monitor their biodistribution in mice using single-photon emission computed tomography (SPECT). The upconversion and the paramagnetic properties of the NaGdF_4_:Yb^3+^/Er^3+^(Tm^3+^)@PEG nanoparticles were evaluated by photoluminescence, magnetic resonance (MR) relaxometry, and magnetic resonance imaging (MRI) with 1 T and 4.7 T preclinical scanners. MRI data were obtained on phantoms with different particle concentrations and during pilot long-time in vivo observations of a mouse model. The biological and physicochemical properties of the NaGdF_4_:Yb^3+^/Er^3+^(Tm^3+^)@PEG nanoparticles make them promising as a trimodal optical/MRI/SPECT bioimaging and theranostic nanoprobe for experimental medicine.

## Introduction

The development of sensitive imaging and therapeutic probes that allow for facile diagnosis, diagnosis-driven treatment, and treatment monitoring of various diseases have recently attracted a great deal of attention^1,2^. Among the probes, nanometer-sized particles in particular have been extensively utilized as contrast agents for anatomical and functional in vivo imaging^3^. Many imaging techniques, such as magnetic resonance imaging (MRI), X-ray computed tomography (CT), single photon emission computed tomography (SPECT), positron emission tomography (PET), ultrasound, optical imaging, and magnetic particle imaging (MPI) have been successfully used in preclinical research for noninvasive real-time monitoring of nanoprobe biodistribution in experimental animals^4–6^. Each of these methods has its own advantages and drawbacks. MRI is considered to be an excellent anatomical and functional modality that provides high spatial resolution without depth limitations and ionization radiation; however, it is limited by lower sensitivity that results in poor contrast and relatively long scanning times^7^. In vivo optical imaging is compromised by poor penetration of the light into the tissue, which does not allow for quantitative analysis. To optimize imaging sensitivity, contrast probes should preferably emit light in the red or near-infrared (NIR) region (~ 600–1000 nm)^6,8^. CT provides a superb hard tissue contrast; however, it has a low soft tissue contrast and produces harmful ionizing radiation^9^. As a result, no single imaging modality is able to provide thorough preclinical information, making it difficult to obtain precise disease diagnoses. Thus, merging several imaging modalities into an “all-in-one” system can tremendously improve sensitivity and provide the better resolution needed for the detection of diseases, while overcoming limitations of a single modality alone^10^.

Lanthanide-based upconversion nanoparticles (UCNPs) have recently attracted extensive scientific interest due to their unique photoluminescence properties^11–14^. UCNPs are able to upconvert low-energy NIR irradiation into high-energy visible or ultraviolet (UV) light via anti-Stokes emission. The ability to absorb NIR radiation allows for deep tissue penetration due to reduced light absorption in the tissue, lower autofluorescence and photon scattering^15^. Compared to conventional fluorescence nanomaterials (e.g., organic dyes or metal complexes), UCNPs have several advantages, such as high chemical stability, large anti-Stokes shift, narrow emission lines, no blinking, and no bleaching^16^. UCNPs have great potential in a variety of applications, from photovoltaics, photocatalysis, security, and display technology to in vitro and in vivo tissue imaging, background-free biosensors, light-triggered drug and gene delivery, optogenetics, photodynamics and photothermal therapy^17–22^. The nanoparticles are also beneficial for multimodal in vivo bioimaging because simple variations of dopant ions in the crystal lattice can induce the formation of particles suitable for down- and upconversion luminescence, MRI, and CT imaging^23,24^. As an example, UCNPs containing Gd^3+^ ions in the host lattice exhibit a short longitudinal relaxation time *T*_1_, which makes their prospective use for luminescent/MR imaging very attractive^25^.

Despite huge progress in the synthesis and understanding of the properties of UCNPs during recent years^26,27^, a number of limitations still have to be solved before these unique particles are translated into clinical praxis. One of the main challenges is their poor colloidal stability in water, and more importantly, in physiological buffers (e.g., PBS). High-quality monodispersed UCNPs with size < 50 nm are typically prepared in high-boiling point organic solvents in the presence of stabilizers; however, the resulting particles are hydrophobic^28,29^. To make them water-dispersible, different surface modification strategies were utilized, including ligand oxidation, silica encapsulation, coating with polyelectrolytes via a layer-by-layer technique, and ligand exchange with hydrophilic and amphiphilic polymers, e.g., poly(acrylic acid), poly(ethylene glycol) (PEG), polyethyleneimine, poly(vinylpyrrolidone), dextran, chitosan, poly(maleic anhydride-*alt*-1-octadecene), etc.^30–32^. Polymers containing anchoring end-groups, e.g., carboxylic or sulfonic acid, amine, hydroxyl, phosphate or phosphonate groups have also been utilized to modify the UCNP surface^33^. While all of these coating strategies result in the UCNPs being dispersible in water, only coatings based on PEG terminated by bisphosphonate or tetraphosphonate groups proved to produce particles stable in PBS, avoiding aggregation^34,35^. The effectiveness of these coatings originates from a strong binding affinity of the phosphonate groups to the lanthanide ions^36^. Therefore in the present work, highly colloidally stable upconversion and magnetic NaGdF_4_:Yb^3+^/Er^3+^ and NaGdF_4_:Yb^3+^/Tm^3+^ nanoparticles coated by PEG-neridronate were designed as a trimodal contrast agent for photoluminescence, SPECT, and MRI, which was verified on a mouse model.

## Experimental

### Chemicals

Anhydrous gadolinium(III), ytterbium(III), erbium(III) and thulium(III) chlorides (99%), octadec-1-ene (90%), ammonium fluoride, chloramine T hydrate (95%), L-ascorbic acid (99%), and phosphate buffered saline (PBS) were purchased from Sigma-Aldrich (St. Louis, MO, USA). Methoxy poly(ethylene glycol) succinimidyl ester (PEG-NHS; *M*_w_ = 5,000) was received from Rapp Polymere (Tuebingen, Germany). Oleic acid (OA), hexane, methanol, acetone, and dichloromethane were purchased from Lach-Ner (Neratovice, Czech Republic). PD-10 desalting column (Sephadex LH-20) was purchased from Amersham Biosciences (Umeå, Sweden). Na^125^I radiolabeling solution (370 MBq) was obtained from the Institute of Isotopes (Budapest, Hungary). Other chemicals were purchased from Sigma-Aldrich and used as received. Cellulose dialysis membranes (3.5 and 14 kDa) were obtained from Spectrum Europe (Breda, Netherlands). AlamarBlue was purchased from Thermo Fisher Scientific (Waltham, MA, USA). Sodium neridronate, (6-amino-1-hydroxy-1-phosphono-hexyl)phosphonic acid sodium salt, Bolton-Hunter reagent (*N*-succinimidyl-3-(4-hydroxyphenyl)propionate), and Bolton-Hunter-neridronate (BH-Ner) were synthesized according to previous publications with minor modifications^35,37,38^. Ultrapure Q-water ultrafiltered on a Milli-Q Gradient A10 system (Millipore; Molsheim, France) was used in the experiments.

### Synthesis of NaGdF_4_:Yb^3+^/Er^3+^ and NaGdF_4_:Yb^3+^/Tm^3+^ nanoparticles

NaGdF_4_:Yb^3+^/Er^3+^ or NaGdF_4_:Yb^3+^/Tm^3+^ nanoparticles were synthesized according to a previously published procedure^39^. Typically, GdCl_3_ (0.78 mmol), YbCl_3_ (0.2 mmol), ErCl_3_ or TmCl_3_ (0.02 mmol), OA (6 ml), and octadec-1-ene (15 ml) were heated at 160 °C for 30 min under argon atmosphere to get a yellowish solution, which was cooled to room temperature (RT), NaOH (2.5 mmol) and NH_4_F (4 mmol) in methanol (5 ml) were added and the mixture was slowly heated to 70 °C under a gentle flow of argon until methanol was evaporated. The mixture was then heated at 300 °C for 90 min, cooled to RT, the particles were precipitated by acetone (10 ml), separated using a centrifuge, washed with hexane, ethanol, ethanol/water mixture (five time each), and 0.01 M HCl, and dialyzed using a cellulose dialysis membrane (14 kDa) against water at RT for 48 h.

### Synthesis of PEG-neridronate (PEG-Ner)

PEG-neridronate (PEG-Ner) was prepared according to previously published report^35^. In a typical procedure, sodium neridronate (0.32 g; 1 mmol) was dissolved in 0.1 M PBS (10 ml; pH 7.4), the solution was cooled to 5 °C, PEG-NHS (1 g; 0.1 mmol of NHS groups) was added, the mixture was stirred at 5 °C for 6 h, dialyzed against water at RT for 48 h, and freeze-dried. The degree substitution of NHS end-groups of PEG with neridronate calculated from ^1^H NMR corresponded to 54%.

### PEGylation of NaGdF_4_:Yb^3+^/Er^3+^ and NaGdF_4_:Yb^3+^/Tm^3+^ nanoparticles

To an aqueous dispersion of NaGdF_4_:Yb^3+^/Er^3+^ or NaGdF_4_:Yb^3+^/Tm^3+^ nanoparticles (5 ml; 5.5 mg/ml), PEG-Ner (0.04 g) was added and the mixture was stirred for 24 h. To remove unreacted PEG-Ner, the resulting NaGdF_4_:Yb^3+^/Er^3+^@PEG or NaGdF_4_:Yb^3+^/Tm^3+^@PEG particles were dialyzed using a cellulose membrane (14 kDa) against water at RT for 72 h.

### Synthesis of NaGdF_4_:Yb^3+^/Er^3+^@PEG-BH-Ner nanoparticles

BH-Ner (0.6 mg) was added to aqueous NaGdF_4_:Yb^3+^/Er^3+^@PEG nanoparticle dispersion (3 ml; 4 mg/ml) and the mixture was stirred at RT for 24 h. To remove residual BH-Ner, resulting NaGdF_4_:Yb^3+^/Er^3+^@PEG-BH-Ner nanoparticles were dialyzed using a cellulose membrane (14 kDa) against water at RT for 48 h and filtered through a Millex-HA syringe filter (0.45 μm pore size). Concentration of the nanoparticles was 4 mg/ml (determined after drying by gravimetric analysis).

### Synthesis of ^125^I-labeled NaGdF_4_:Yb^3+^/Er^3+^@PEG nanoparticles

NaGdF_4_:Yb^3+^/Er^3+^@PEG-BH-Ner particles were radiolabeled with ^125^I using chloramine T with minor modifications^35,40^. Typically, NaGdF_4_:Yb^3+^/Er^3+^@PEG-BH-Ner nanoparticle dispersion (200 µl; 4 mg/ml), radiolabeling Na^125^I solution (3 µl; 9.8 MBq), and chloramine T (10 µl; 10 mg/ml of PBS) were mixed in 0.1 M PBS (100 µl; pH 7.4) at RT for 3 h with shaking; a solution of ascorbic acid in 0.1 M PBS (100 µl; 25 mg/ml) was added and the reaction continued at RT for 18 h. To remove low-molecular-weight residues and unreacted ^125^I, the resulting NaGdF_4_:Yb^3+^/Er^3+^@PEG-^125^I nanoparticles were separated on a PD-10 desalting column with 0.1 M PBS as a mobile phase; altogether 20 fractions (0.75 ml each) were collected. Radioactivity of the particle dispersion was analyzed by a Bqmetr 4 ionization chamber (Empos; Prague, Czech Republic).

### Characterization methods

The size, composition, and crystal structure of the nanoparticles was analyzed using a Tecnai Spirit G2 transmission electron microscope (TEM; FEI; Brno, Czech Republic)^39^. The particles were deposited on a standard carbon-coated copper grid and visualized by means of bright field imaging at 120 kV. The microscope was equipped with an energy dispersive spectrometer (EDX; Mahwah, NJ, USA) to analyze elements in the nanoparticles and selected area electron diffraction (SAED) mode to verify the crystal structure^39^. The electron diffraction patterns were processed with ProcessDiffraction software^41^ and compared with the diffraction patterns calculated with PowderCell software^42^; the crystal structures for the diffraction pattern calculation were obtained from ICSD database^43^. Particle size and distribution was determined by measuring at least 300 nanoparticles from four randomly selected TEM micrographs using Atlas software (Tescan; Brno, Czech Republic)^44^. Number- (*D*_n_), weight-average diameter (*D*_w_), and the uniformity (dispersity  ) were calculated as follows^39^:

1 $$D_{{\text{n}}} = \sum {\text{N}}_{{\text{i}}} D_{{\text{i}}} /\sum {\text{N}}_{{\text{i}}} ,$$

2 $$D_{{\text{w}}} = \sum {\text{N}}_{{\text{i}}} D_{{\text{i}}}^{4} /\sum {\text{N}}_{{\text{i}}} D_{{\text{i}}}^{3} ,$$

3 where N_i_ and *D*_i_ are number and diameter of the nanoparticle, respectively.

Hydrodynamic particle diameter (*D*_h_), size distribution (polydispersity *PD*), and ζ-potential were analyzed by a dynamic light scattering (DLS) on a ZEN 3600 Zetasizer Nano Instrument (Malvern Instruments; Malvern, UK)^39^. The nanoparticle dispersion was measured at 25 °C, and the *D*_h_ and *PD* were calculated from the intensity-weighted distribution function obtained by CONTIN analysis of the correlation function embedded in Malvern software.

Thermogravimetric analysis (TGA) was performed from 30 to 600 °C using a Perkin Elmer TGA 7 analyzer (Norwalk, CT, USA) at a heating rate of 10 °C/min under air atmosphere^45^.

ATR FTIR spectra were recorded on a Nexus Nicolet 870 FTIR spectrometer (Madison, WI, USA) equipped with a liquid nitrogen cooled mercury cadmium telluride detector. The spectra were measured using a Golden Gate single reflection ATR cell (Specac; Orpington, UK) equipped with a diamond internal reflection ATR crystal^39^.

Inductively coupled plasma mass spectrometry (ICP-MS) was performed on a mass spectrometer with an inductively coupled plasma Elan DRC-e (Perkin Elmer; Concord, Canada); ^158^Gd isotope was measured with ^103^Rh as an inner standard.

### Upconversion luminescence and single nanoparticle imaging

For the steady state photoluminescence, a 980-nm laser (Shanghai Dream Lasers Technology SDL-980-LM-1000 T) was used as an excitation source. An HR4000 spectrometer (Ocean Optics; Ostfildern, Germany) was used as a detection system for measurements in a visible spectral range. The emission spectra were not corrected spectrally. Photoluminescence decays were measured using an Opolette pulsed laser (Opotek; Carlsbad, CA, USA) at 978 nm, 7 ns, 20 Hz, and average optical power P_av_ = 3 mW. The laser was coupled to a gated detection system with a time resolution of 1 μs. Single UCNPs were imaged using a custom-build wide-field fluorescence microscope equipped with 980 nm CW laser (CNI; MDL-980), EMCCD (Cascade 512B; Photometrics; Buckinghamshire, UK), and oil immersion objective (Olympus; Tokyo, Japan; 100 × , NA 1.3). A highly diluted UCNP dispersion in hexane was drop-casted on the microscope coverslip and left for solvent evaporation. Sample was placed on the motorized XYZ stage with closed-loop piezo actuators (Thorlabs; Newton, NJ, USA). The luminescence light was extracted with a short-pass dichroic beamsplitter followed by a dual-band bandpass filter (Semrock; Lake Forest, IL, USA).

### Magnetic resonance

*Magnetic resonance imaging* (MRI) was performed on phantoms containing particles at 25 °C on a 4.7 T Bruker Biospec spectrometer equipped with a commercially available resonator coil. Standard two-dimensional rapid acquisition with a relaxation enhancement multispin echo (RARE) sequence was used with the following parameters: repetition time 280 ms, echo time 12 ms, turbo factor 2, spatial resolution 137 × 137 mm^2^, slice thickness 0.7 mm, number of acquisitions 16, and acquisition time 9 min 36 s. The signal-to-noise ratio (SNR) was 0.655∙S/σ, where S is signal intensity in a region of interest, σ is the standard deviation of the noise in background, and constant 0.655 reflects Rician distribution of the background noise in a magnitude MR image.

*Magnetic resonance relaxometry* of NaGdF_4_:Yb^3+^/Er^3+^(Tm^3+^) particles with and without PEG-neridronate modification was performed on a Minispec mq60 (Bruker; Germany) at 37 °C and magnetic field B_0_ = 1.41 T. The experimentally determined solvent relaxation rate *R* (concentration 0 mM) was subtracted as a starting value from the nanoparticle relaxation rates prior to the linear regression analysis. Summary of relaxation results are shown in Tables S1 and S2.

### Cytotoxicity of the particles

Cytotoxicity of the NaGdF_4_:Yb^3+^/Er^3+^ and NaGdF_4_:Yb^3+^/Er^3+^@PEG nanoparticles was evaluated on a cell line derived from human cervical carcinoma (HeLa) and human dermal fibroblast (HF)^46–48^ kindly provided by Dr. Mělková and Dr. Dvořánková, respectively, First Faculty of Medicine, Charles University, Prague. 5∙10^3^ of HeLa or 8∙10^3^ of HF cells were seeded in 100 µl of media into 96-well flat-bottom TPP plates (Merck; Darmstadt, Germany) for 24 h. Subsequently, the nanoparticles (0.4–400 µg/ml) were added, the cells were cultivated at 37 °C for 72 h under 5% CO_2_ atmosphere, AlamarBlue cell viability reagent (10 µl) was added to each well, and the incubation continued at 37 °C for 4 h. The active component of the AlamarBlue reagent (resazurin) was reduced to the highly fluorescent resorufin only in viable cells. Fluorescence was detected using a Synergy Neo plate reader (Bio-Tek; Winooski, VT, USA) at 570 nm (excitation) and 600 nm (emission). Cells cultivated in medium without the nanoparticles served as a control. The assay was repeated two to three times in tetraplicates.

### Experimental animals

Four 12 weeks old female adult BALB/C mice (Charles River Laboratories; Erkrath, Germany) were maintained in individually ventilated cages (12:12 h light–dark cycle, 22 ± 1 °C, 60 ± 5% humidity) with standard food and access to water ad libitum. Animals were regularly observed during the whole experiment for changes in their behavior and health status. First two mice were measured on an Albira SPECT/PET/CT imaging system (Bruker BioSpin; Ettlingen, Germany), while the next two mice were imagined on MRI preclinical scanner (ICON; Bruker BioSpin).

All in vivo experiments were performed in accordance with national and international guidelines for laboratory animal care and approved by the Laboratory Animal Care and Use Committee of the First Faculty of Medicine, Charles University in Prague and the Ministry of Education, Youth and Sports of the Czech Republic (MSMT-6316/2014–46).

### Biodistribution of the NaGdF_4_:Yb^3+^/Er^3+^@PEG-^125^I nanoparticles in mice on SPECT/CT

Dispersion of the NaGdF_4_:Yb^3+^/Er^3+^@PEG-^125^I nanoparticles in PBS buffer (200 µl; 30 MBq/mouse) was intravenously injected into two mice via the tail vein. ^125^I with a long half-life (59.49 days) was selected as a radioisotope because it is cheap, easily available on the market, readily chemically modifiable, and very suitable especially for small-animal SPECT with an Albira imaging system used in our work. The low-energy emission (35 keV) of ^125^I is also a great advantage for the longitudinal studies. Biodistribution of the particles in mice was evaluated under anesthesia (2% isoflurane in air); both mice were scanned at 30, 50, 70, and 260 min and 1, 2, 6, and 8 days after the injection and acquisitions were obtained using multi-pinhole collimators. Acquisition time was 45 min (90 s/projection) and followed by a single CT scan (15 min). Image analysis and co-registration was done using PMOD analysis software (PMOD Technologies; Zürich, Switzerland).

### Biodistribution of the NaGdF_4_:Yb^3+^/Er^3+^@PEG nanoparticles in mice on MRI

NaGdF_4_:Yb^3+^/Er^3+^@PEG nanoparticles in PBS buffer (200 µl; 4 mg/ml) were intravenously injected into two mice and ICON imager with magnetic field 1 T was used for MRI imaging. Biodistribution of the nanoparticles was determined under anesthesia (2% isoflurane in air) with monitoring of vital functions (breathing and cardiac rate). The imaging parameters were following: *T*_1_ RARE sequence, repetition time/echo time 350/12 ms, thickness 1 mm, coronal orientation, matrix 256 × 256 pixels, field of view 90 × 35 mm, 8 averages, duration 4.5 min. Image and data analysis was performed using ParaVision 6.0 software (Bruker BioSpin).

## Results

### NaGdF4:Yb^3+^/Er^3+^(Tm^3+^) nanoparticles

**Figure 1:**
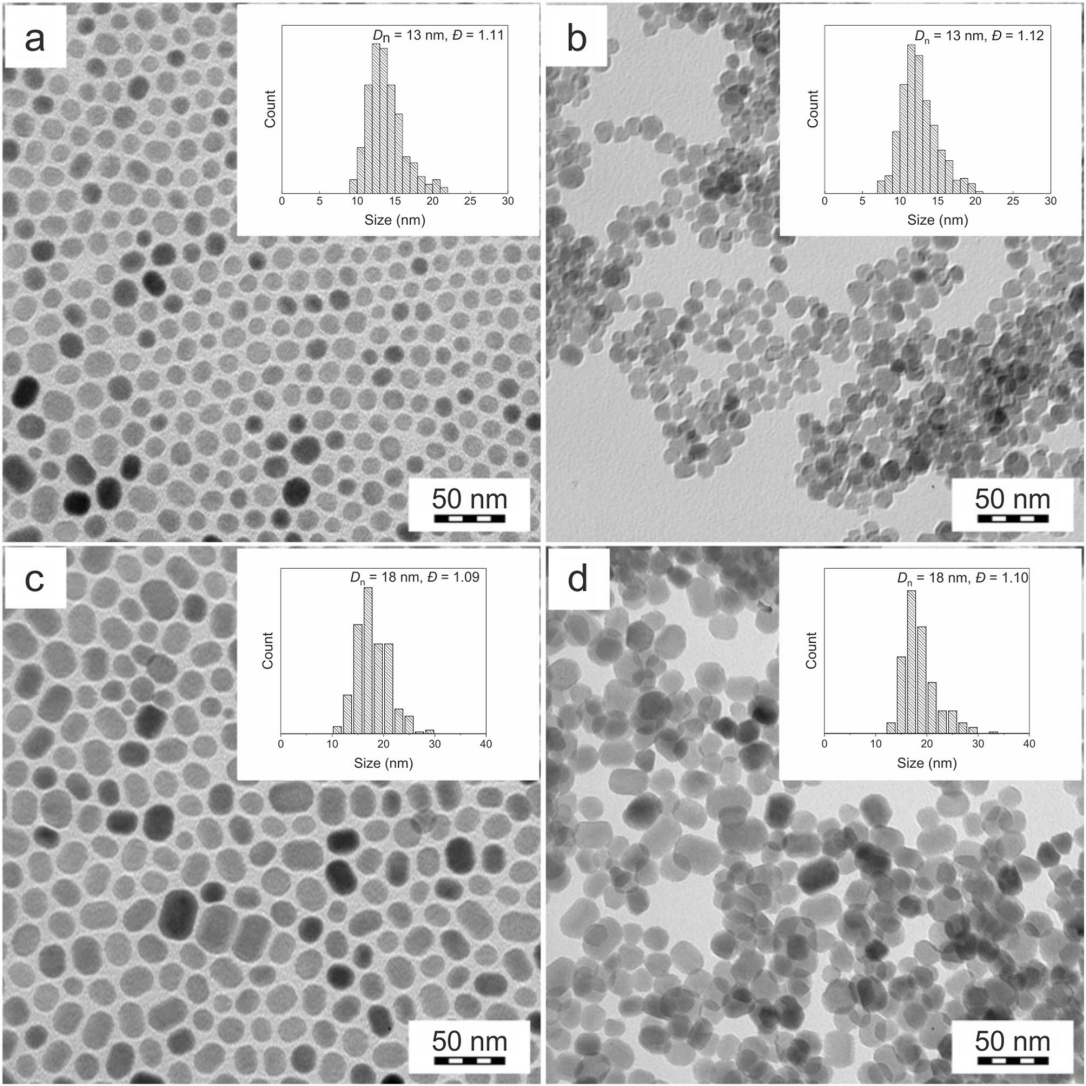
TEM micrographs of (**a**) OA-NaGdF_4_:Yb^3+^/Er^3+^ and (**c**) OA-NaGdF_4_:Yb^3+^/Tm^3+^ nanoparticles in hexane and (**b**) NaGdF_4_:Yb^3+^/Er^3+^@PEG and (**d**) NaGdF_4_:Yb^3+^/Tm^3+^@PEG nanoparticles in water.

Upconversion and magnetic NaGdF_4_:Yb^3+^/Er^3+^ and NaGdF_4_:Yb^3+^/Tm^3+^ nanoparticles were synthesized by a high-temperature coprecipitation of lanthanide precursors in octadec-1-ene in the presence of oleic acid (OA), which stabilized the particles during their nucleation and growth. According to TEM, the size of the OA-NaGdF_4_:Yb^3+^/Er^3+^ and OA-NaGdF_4_:Yb^3+^/Tm^3+^ particles was *D*_n_ = 13 and 18 nm, respectively, with a relatively narrow particle size distribution  = 1.11 and 1.09, respectively (Fig. 1a,c).

The crystal structure and elemental composition of the nanoparticles was verified by TEM microscopy using selected area electron diffraction (SAED) and energy-dispersive analysis of X-rays (EDX). The SAED diffractogram corresponded to the hexagonal phase of NaGdF_4_, as confirmed by the transformation of the experimental 2D diffraction pattern (Electronic supplementary information, Fig. S1a) to a 1D diffractogram that was compared to the theoretically calculated 1D X-ray diffractogram (XRD) of hexagonal NaGdF_4_ (Fig. S1b)^43^. The agreement between the experimental and theoretically calculated diffractograms was very good. The positions of the four most intense diffraction rings corresponded exactly to the strongest diffractions of NaGdF_4_ (Fig. S1a), and the same was observed for the less intense diffraction peaks (Fig. S1b). The small differences between the XRD and SAED diffraction intensities could be attributed to the following three facts: (*i*) the first SAED ring was partially masked by the strong primary beam, (*ii*) the second SAED maximum (corresponding to a pair of XRD diffractions) was used for normalization, and (*iii*) the remaining SAED intensities were somewhat lower than those of the corresponding XRD diffractions probably due to the intrinsic differences of the two methods and/or possible preferred orientation effects^45^.

An elemental analysis of the NaGdF_4_:Yb^3+^/Er^3+^ nanoparticles with TEM/EDX confirmed the electron diffraction results (Fig. S1c). Except for the peaks corresponding to the carbon-coated copper grid (C, Cu), the strongest peaks (Na, F, Gd, and Yb) in the EDX spectrum corresponded to the expected composition of the prepared NaGdF_4_:Yb^3+^/Er^3+^ nanocrystals. Erbium peaks (expected at positions Mα = 1.4 keV and Lα = 6.9 keV) could not be detected due to a negligible amount of Er and possible overlaps with the stronger Gd and Yb peaks at given energies.

### Surface modification of NaGdF_4_:Yb^3+^/Er^3+^(Tm^3+^) nanoparticles with poly(ethylene glycol) (PEG)

The OA-stabilized upconversion nanoparticles were hydrophobic and therefore not dispersible in aqueous media. However, thorough washing of the particles with hexane, ethanol, an ethanol/water mixture, and treatment with 0.01 M HCl made them dispersible and colloidally stable in water (Fig. S2a). The hydrodynamic diameter of the NaGdF_4_:Yb^3+^/Er^3+^ nanoparticles in water was *D*_h_ = 151 nm and *PD* = 0.22 with highly positive charge (ζ-potential = 41 mV) due to the presence of lanthanide ions on the particle surface; however, the particles aggregated in PBS. After modification with PEG-Ner, the number-average diameter did not change (Fig. 1b,d). Compared to pure NaGdF_4_:Yb^3+^/Er^3+^ nanoparticles, ζ-potential of the NaGdF_4_:Yb^3+^/Er^3+^@PEG particles significantly decreased (10 mV) due to highly electroneutral and hydrophilic nature of PEG. The hydrodynamic diameter of the NaGdF_4_:Yb^3+^/Er^3+^@PEG nanoparticles in PBS reached ~ 100–110 nm and did not change with time (Fig. S3a illustrates only the first 11 days, but the particles did not aggregate within a month). In addition, the polydispersity *PD* of ~ 0.2 remained constant, showing a narrow size distribution.

The NaGdF_4_:Yb^3+^/Er^3+^, OA-NaGdF_4_:Yb^3+^/Er^3+^, and NaGdF_4_:Yb^3+^/Er^3+^@PEG nanoparticles were also characterized by thermogravimetric analysis (TGA) in the range of 40–600 °C (Fig. S3b); the corresponding first derivatives of the TGA curves are shown in Fig. S4. OA and PEG-Ner completely decomposed at ~ 300–420 °C and 200–260 °C, respectively (Fig. S4a,b). The weight loss in the OA-NaGdF_4_:Yb^3+^/Er^3+^ particles was relatively low at 200–350 °C but higher at ~ 350–480 °C (Fig. S4c), reaching 6.6 wt.%, which was attributed to the combustion of OA. The rather low weight loss in the thermogram of the NaGdF_4_:Yb^3+^/Er^3+^ particles (1.8 wt.%) can be ascribed to the residual OA remaining after washing (Fig. S4d). In the thermogram of the NaGdF_4_:Yb^3+^/Er^3+^@PEG particles, the weight loss occurred at 200–470 °C, which was ascribed to the decomposition of PEG (Fig. S4e). It can be thus assumed that the NaGdF_4_:Yb^3+^/Er^3+^@PEG particles contained 19.6 wt.% of PEG (Fig. S3b).

The final physicochemical characterization of the OA-NaGdF_4_:Yb^3+^/Er^3+^, NaGdF_4_:Yb^3+^/Er^3+^, and NaGdF_4_:Yb^3+^/Er^3+^@PEG nanoparticles involved ATR FTIR spectroscopy (Fig. S3c). The spectrum for the freshly prepared OA-NaGdF_4_:Yb^3+^/Er^3+^ nanoparticles contained the characteristic OA bands, i.e., asymmetric and symmetric stretching vibrations of CH_2_ groups at 2,925 and 2,853 cm^−1^, stretching vibrational mode of C=O of COOH groups in OA at 1,712 cm^−1^, bending vibrations of CH_2_ groups at 1,462 cm^−1^, and out-of-plane bending of C–OH at 1,411 cm^−1^^49, 50^. The spectrum for the NaGdF_4_:Yb^3+^/Er^3+^ nanoparticles confirmed the efficacy of washing, as the intensity of the bands for OA significantly decreased. As expected, the particle surface modification with PEG-Ner changed the spectrum, and the characteristic bands of PEG at 2,885, 1,342, 1,280, 1,241, 1,106, 963, and 844 cm^−1^ appeared. The band at 2,885 cm^−1^ was assigned to symmetric stretching of CH_2_ groups, the band at 1,342 cm^−1^ to wagging vibrations of CH_2_ groups, the bands at 1,280 and 1,241 cm^−1^ to twisting vibrations of CH_2_ groups of PEG chains that adopted helical and *trans* planar structures, the band at 1,106 cm^−1^ to coupled C–O and C–C stretching vibrations of PEG backbone, and the bands at 963 and 844 cm^−1^ to rocking vibrations of CH_2_ groups of PEG when these chains adopted *trans* planar and helical structures^51,52^. The bands were strong, with intensities comparable to those in the spectrum of neat PEG^51^. ATR FTIR spectroscopy thus confirmed the successful modification of the NaGdF_4_:Yb^3+^/Er^3+^ nanoparticles with PEG, which was in agreement with results from the other experimental methods used in this work.

### Cytototoxicity of NaGdF_4_:Yb^3+^/Er^3+^ and NaGdF_4_:Yb^3+^/Er^3+^@PEG nanoparticles

The cytotoxicity of the particles was determined on HeLa cells and primary fibroblasts (HF) using an AlamarBlue viability assay. The nanoparticles did not cause toxicity in HeLa cells, the viability of which did not go below 90% (Fig. S5a). At the two highest particle concentrations (200 and 400 μg/ml), a slight decrease of HF cell viability was observed; however, it did not decline to < 80% (Fig. S5b). At the highest concentration, the NaGdF_4_:Yb^3+^/Er^3+^@PEG nanoparticles showed significantly lower toxicity than those without PEG, reflecting their high biocompatibility.

### Upconversion photoluminescence of NaGdF_4_:Yb^3+^/Er^3+^(Tm^3+^) nanoparticles

**Figure 2:**
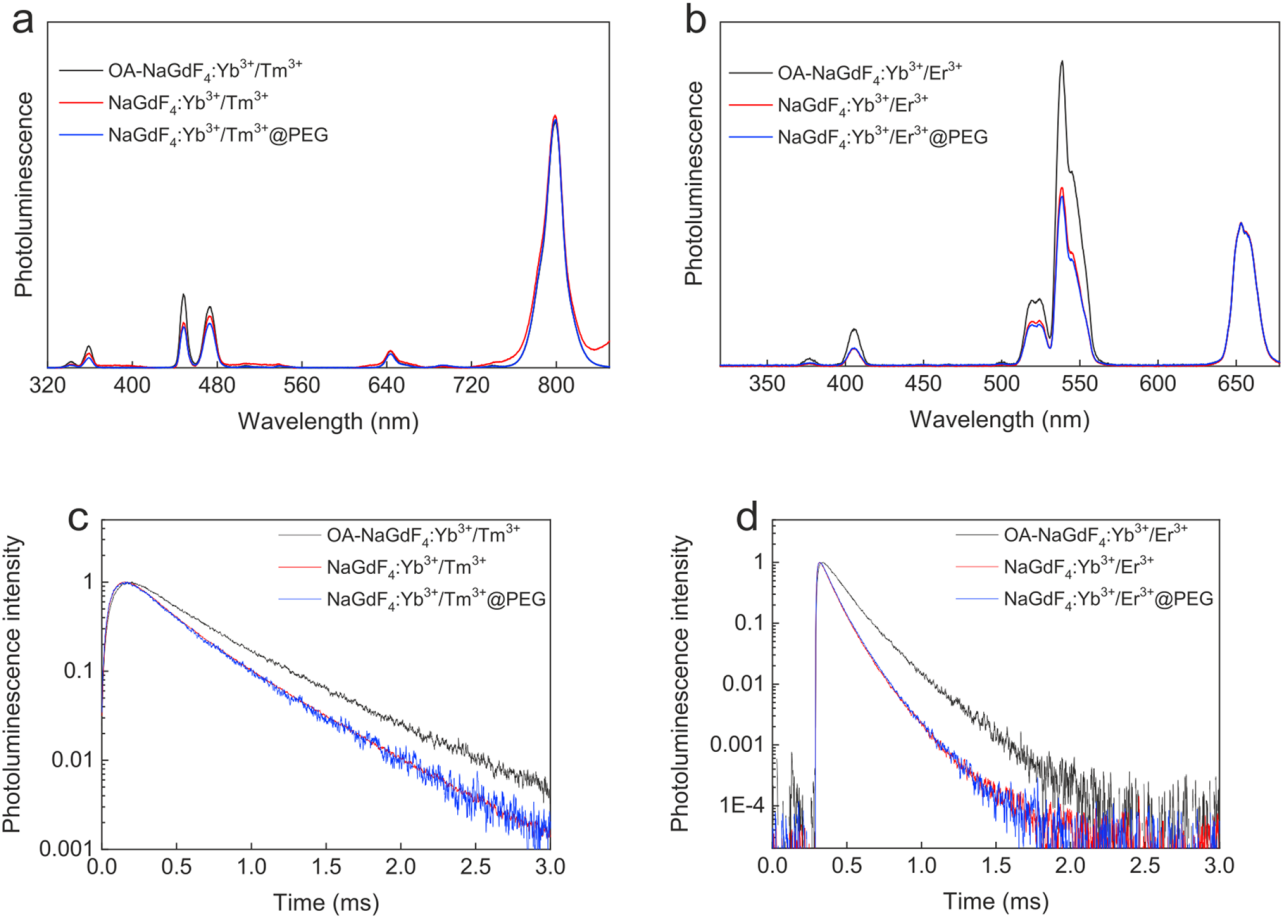
(**a**,**b**) Normalized upconversion photoluminescence emission spectra of (**a**) NaGdF_4_:Yb^3+^/Tm^3+^- and (**b**) NaGdF_4_:Yb^3+^/Er^3+^-based particles; not corrected by the spectral response of the detection system, nominal laser power P = 150 mW, and excitation at 980 nm. (**c**,**d**) Upconversion emission decay time profiles of (**c**) NaGdF_4_:Yb^3+^/Tm^3+^- and (**d**) NaGdF_4_:Yb^3+^/Er^3+^-based particles; emission at (**c**) 800 and (**d**) 540 nm, excitation at 978 nm (7 ns pulse duration, P_av_ = 1 mW).

The photoluminescence spectra of colloidal NaGdF_4_:Yb^3+^/Tm^3+^ nanoparticles at 980 nm diode laser excitation (nominal laser power on the sample P = 150 mW; power density 7.5 kW/cm^2^) showed the main bands at 450, 480, and 800 nm (Fig. 2a), corresponding to the ^1^D_2_ → ^3^F_4_, ^1^G_4_ → ^3^H_6_, and ^3^H_4_ → ^3^H_6_ transitions of the Tm^3+^ ions, respectively. Much lower intensity bands were observed at 330, 366, and 650 nm, corresponding to the ^1^I_6_ → ^3^F_4_, ^1^D_2_ → ^3^H_6_, and ^1^G_4_ → ^3^F_4_ transitions, respectively. The emission spectra of the NaGdF_4_:Yb^3+^/Er^3+^ particles recorded under the same experimental conditions showed typical Er^3+^ emission lines at 375 nm (^4^G_11/2_ $\to$ ^4^I_15/2_), 520 nm (^2^H_11/2_ $\to$ ^4^I_15/2_), 540 nm (^4^S_3/2_ $\to$ ^4^I_15/2_), 650 nm (^4^F_9/2_ $\to$ ^4^I_15/2_), and 840 nm (^4^I_9/2_ $\to$ ^4^I_15/2_); moreover, there was a strong blue band at 410 nm (Fig. 2b). This emission could be related to ^2^H_9/2_ $\to$ ^4^I_15/2_ transition in the Er^3+^ ions. Only a small difference was observed in the NaGdF_4_:Yb^3+^/Tm^3+^ and NaGdF_4_:Yb^3+^/Er^3+^ luminescence spectra on the low energy side of the spectrum after the nanocrystal surface modification. Since the particles were relatively small (18 nm), the surface Tm^3+^ ions could have a visible contribution to the total emission intensity^53,54^. Thus, the surface modifications changed the surface ion properties by varying excited carrier relaxation processes. These alterations were visible in the relative change of emission intensities. In the luminescence spectrum of the NaGdF_4_:Yb^3+^/Tm^3+^@PEG particles on the high energy level, only a small difference was found compared to the spectrum of the unmodified particles (Fig. 2a,b). In contrast, this change was much more significant in the NaGdF_4_:Yb^3+^/Er^3+^-based nanoparticles. This could be explained by different positions of energy levels of the ions relative to those of organic modifying agents on the particle surface. Other effect that should be considered is associated with spectrally dependent absorption of water and/or hexane, resulting in different quenching of the emitted light depending on the spectral range.

To better understand the optical properties of the NaGdF_4_:Yb^3+^/Tm^3+^ and NaGdF_4_:Yb^3+^/Er^3+^ nanoparticles, emission decay traces of the surface-modified particles have also been recorded (Fig. 2c,d) and the average values of the decay times have been recorded. To analyze the results, the simplest approach was used and for all the decay curves an average decay time $\left\langle \tau \right\rangle$ was calculated according to the Eq. (4):

4 $$\left\langle \tau \right\rangle = \frac{{\mathop \smallint \nolimits_{to}^{t1} t \cdot I\left( t \right)dt}}{{\mathop \smallint \nolimits_{to}^{t1} I\left( t \right)dt}},$$

where *I*(*t*) represents the photoluminescence intensity at time *t* after cutoff of the excitation light, *t*_0_ = 0 s is the initial time, when the signal starts to decay, and *t*_1_ is the time, when the luminescence intensity reaches the background. The average decay time was longer for the NaGdF_4_:Yb^3+^/Tm^3+^ nanoparticles (τ_800_ = 340 μs) than for the NaGdF_4_:Yb^3+^/Er^3+^ nanoparticles (τ_540_ = 180 μs). In addition, the recombination process for the Er^3+^ ions was much less exponential compared to the recombination for the Tm^3+^ ions. At the same time, a small decrease in the emission decay time of the NaGdF_4_:Yb^3+^/Tm^3+^(Er^3+^) and NaGdF_4_:Yb^3+^/Tm^3+^(Er^3+^)@PEG nanoparticles compared to the OA-NaGdF_4_:Yb^3+^/Tm^3+^(Er^3+^) nanoparticles was observed (Fig. 2c,d). This could be because the NaGdF_4_:Yb^3+^/Tm^3+^(Er^3+^) and NaGdF_4_:Yb^3+^/Tm^3+^(Er^3+^)@PEG particles were dispersed in water, while the OA-NaGdF_4_:Yb^3+^/Tm^3+^(Er^3+^) particles were dispersed in hexane. Thus, this small decrease could be due to photonic effects (induced by the surface ions) related to the differences in refractive index for the two solvents (water or hexane). Nevertheless, a more probable explanation for the reduced emission decay time of the NaGdF_4_:Yb^3+^/Tm^3+^(Er^3+^) and NaGdF_4_:Yb^3+^/Tm^3+^(Er^3+^)@PEG particles compared to the OA-NaGdF_4_:Yb^3+^/Tm^3+^(Er^3+^) particles is the appearance of surface defects formed during OA removal or the formation of new ligands, which can introduce new nonradiative channels for the RE^3+^ ions present on the surface.

**Figure 3:**
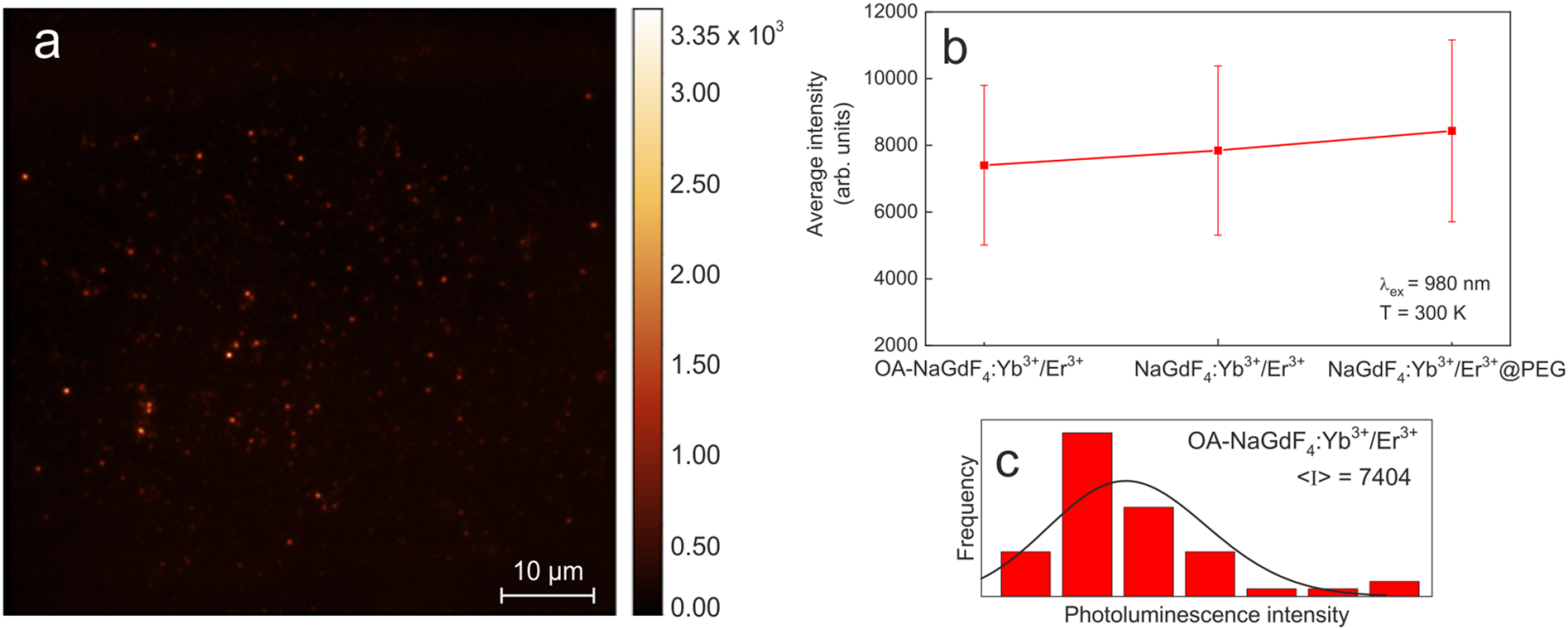
(**a**) An example of fluorescence intensity map of single OA-NaGdF_4_:Yb^3+^/Er^3+^ nanoparticles. (**b**) Average luminescence intensity of various NaGdF_4_:Yb^3+^/Er^3+^-based nanoparticles. (**c**) The distribution of single-particle luminescence intensities. The excitation was at 980 nm. Both green (^2^H_11/2_ → ^4^I_15/2_, ^4^S_3/2_ → ^4^I_15/2_) and red (^4^F_9/2_ → ^4^I_15/2_) Er^3+^ luminescence bands were merged and imaged with a dual-band bandpass filter.

To investigate the effect of surface engineering on the emission intensity, the luminescence intensity maps of single OA-NaGdF_4_:Yb^3+^/Er^3+^, NaGdF_4_:Yb^3+^/Er^3+^, and NaGdF_4_:Yb^3+^/Er^3+^@PEG nanoparticles were measured. In highly diluted colloids, it was possible to spatially separate individual nanoparticles by a distance larger than the point spread function of the optical system. The maps looked very similar, the intensity distribution was similar as well, with only a small difference in average values. As an example, fluorescence intensity map of single OA-NaGdF_4_:Yb^3+^/Er^3+^ nanoparticles was shown on Fig. 3a. The luminescence intensity of the OA-NaGdF_4_:Yb^3+^/Er^3+^ particles in hexane and the NaGdF_4_:Yb^3+^/Er^3+^ and NaGdF_4_:Yb^3+^/Er^3+^@PEG particles in water was determined for fifty single particles each. Based on the histogram of the particle luminescence intensity, its mean value was estimated (Fig. 3b) and the distribution of single-particle luminescence intensities determined (Fig. 3c).

### MR imaging and relaxometry of NaGdF_4_:Yb^3+^/Er^3+^@PEG nanoparticles

**Figure 4:**
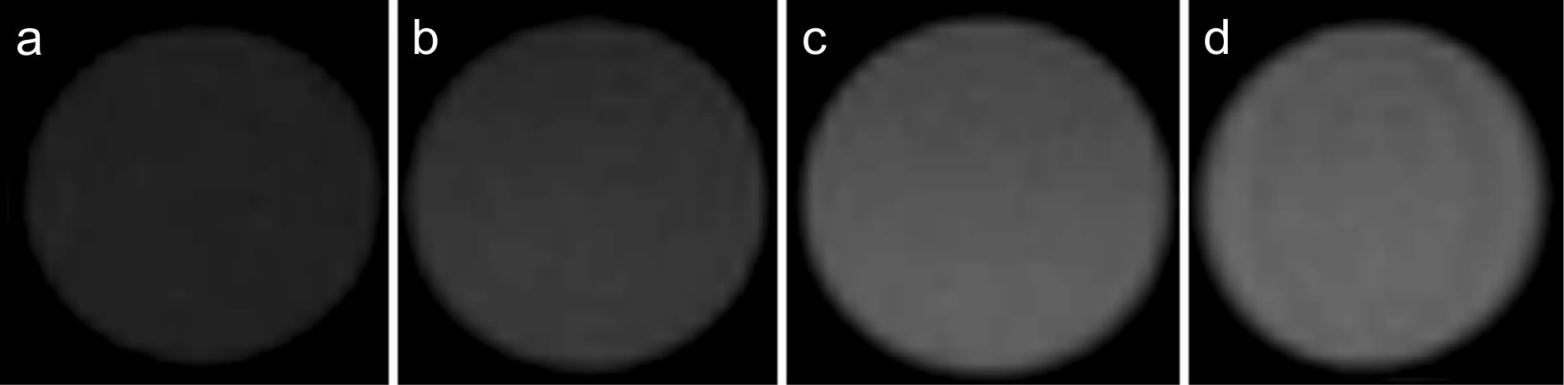
*T*_1_-weighted MR images of NaGdF_4_:Yb^3+^/Er^3+^@PEG particle phantoms measured at 4.7 T using a multispin echo sequence: (**a**) water, (**b**) 0.0027, (**c**) 0.027, and (**d**) 0.27 mM Gd^3+^.

The *T*_1_-weighted MR images of water phantoms containing the NaGdF_4_:Yb^3+^/Er^3+^@PEG nanoparticles proved that the nanoparticles affected the contrast; MR signal was higher with increasing nanoparticle concentration (Fig. 4). The undiluted NaGdF_4_:Yb^3+^/Er^3+^@PEG nanoparticle dispersion (2.7 mM Gd^3+^) decreased the MR signal to the noise level, making the MR image impossible to detect due to the strong paramagnetic effect on the *T*_2_ relaxation at the high particle concentration. The highest signal (for repetition time 280 ms and echo time 12 ms) was observed for the tenfold diluted NaGdF_4_:Yb^3+^/Er^3+^@PEG particle dispersion (0.27 mM Gd^3+^).

For relaxometry study, the concentration of Gd in the NaGdF_4_:Yb^3+^/Er^3+^ and NaGdF_4_:Yb^3+^/Er^3+^@PEG nanoparticle dispersion was determined by inductively coupled plasma mass spectrometry (ICP-MS), reaching 10.9 and 10.7 mM, respectively. Based on these results, relaxivity *r*_1_ and *r*_2_ was calculated by the linear regression analysis (Fig. S6). Results obtained from the imaging phantom study were supported by the relaxometry measurements, where significant shortening of both the *T*_1_ and *T*_2_ relaxation time was observed in NaGdF_4_:Yb^3+^/Er^3+^ and NaGdF_4_:Yb^3+^/Er^3+^@PEG nanoparticles. The *T*_1_ was 163.9 ± 2.1 ms (relaxation rate *R*_1_ = 6.1 s^−1^) for NaGdF_4_:Yb^3+^/Er^3+^ particles at concentration of 10.9 mM Gd^3+^, compared to *T*_1_ = 3,726.0 ± 103.1 ms (*R*_1_ = 0.3 s^−1^) for the control sample of water; the *T*_2_ was 10.4 ± 0.5 ms (relaxation rate *R*_2_ = 96.1 s^−1^), compared to *T*_2_ = 3,440.0 ± 5.6 ms (*R*_2_ = 0.3 s^−1^) for the control sample of water (Table S1).

The *T*_1_ was 95.2 ± 0.8 ms (relaxation rate *R*_1_ = 10.5 s^−1^) for the NaGdF_4_:Yb^3+^/Er^3+^@PEG particles at concentration of 10.7 mM Gd^3+^, compared to *T*_1_ = 3,726.0 ± 103.1 ms (*R*_1_ = 0.3 s^−1^) for the control sample of water; the *T*_2_ was 11.3 ± 0.6 ms (relaxation rate *R*_2_ = 88.5 s^−1^), compared to *T*_2_ = 3,440.0 ± 5.6 ms (*R*_2_ = 0.3 s^−1^) for the control sample of water (Table S2).

Relaxivities for the NaGdF_4_:Yb^3+^/Er^3^ particles were *r*_1_ = 0.560 mM^−1^∙s^−1^ (coefficient of determination *R*^2^ = 0.989) and *r*_2_ = 9.130 mM^−1^∙s^−1^ (*R*^2^ = 0.997; Fig. S6). Relaxivities for the NaGdF_4_:Yb^3+^/Er^3+^@PEG particles were *r*_1_ = 0.964 mM^−1^∙s^−1^ (*R*^2^ = 1.000) and *r*_2_ = 8.271 mM^−1^∙s^−1^ (*R*^2^ = 1.000). The relaxivities *r*_1_ were lower compared to those in published report^55^; however, *r*_2_ were slightly higher than in most commercial contrast agents shown in that reference.

The calculated relaxivity *r*_1_ and *r*_2_ and results obtained from imaging on phantoms suggest that tested MR probes could be successfully used for in vivo MR imaging. Relaxometry study revealed interesting effect of modification with PEG-neridronate. Instead of negligibly decreased *r*_1_ relaxivity of the NaGdF_4_:Yb^3+^/Er^3+^@PEG particles, *r*_1_ was ~ 70% higher compared to that for the unmodified particles. On the other side, relaxivity *r*_2_ of NaGdF_4_:Yb^3+^/Er^3+^@PEG nanoparticles decreased by ~ 10%.

### Biodistribution of NaGdF_4_:Yb^3+^/Er^3+^@PEG and NaGdF_4_:Yb^3+^/Er^3+^@PEG-^125^I nanoparticles

**Figure 5:**
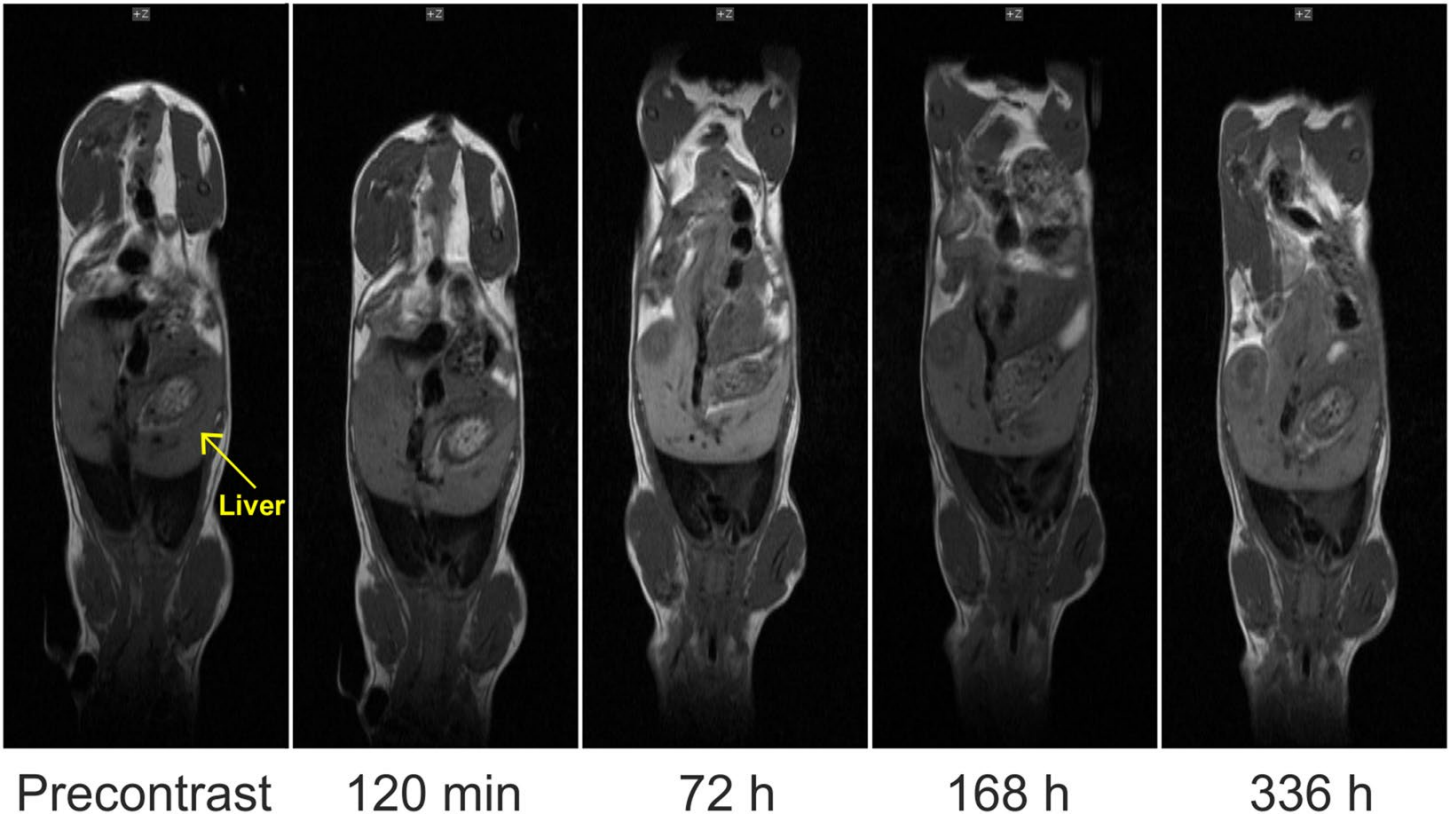
In vivo* T*_1_-weighted MR images of a representative mouse before and during long-time monitoring after the application of NaGdF_4_:Yb^3+^/Er^3+^@PEG nanoparticles (images of the same mouse in coronal orientation) at 1 T.

**Figure 6:**
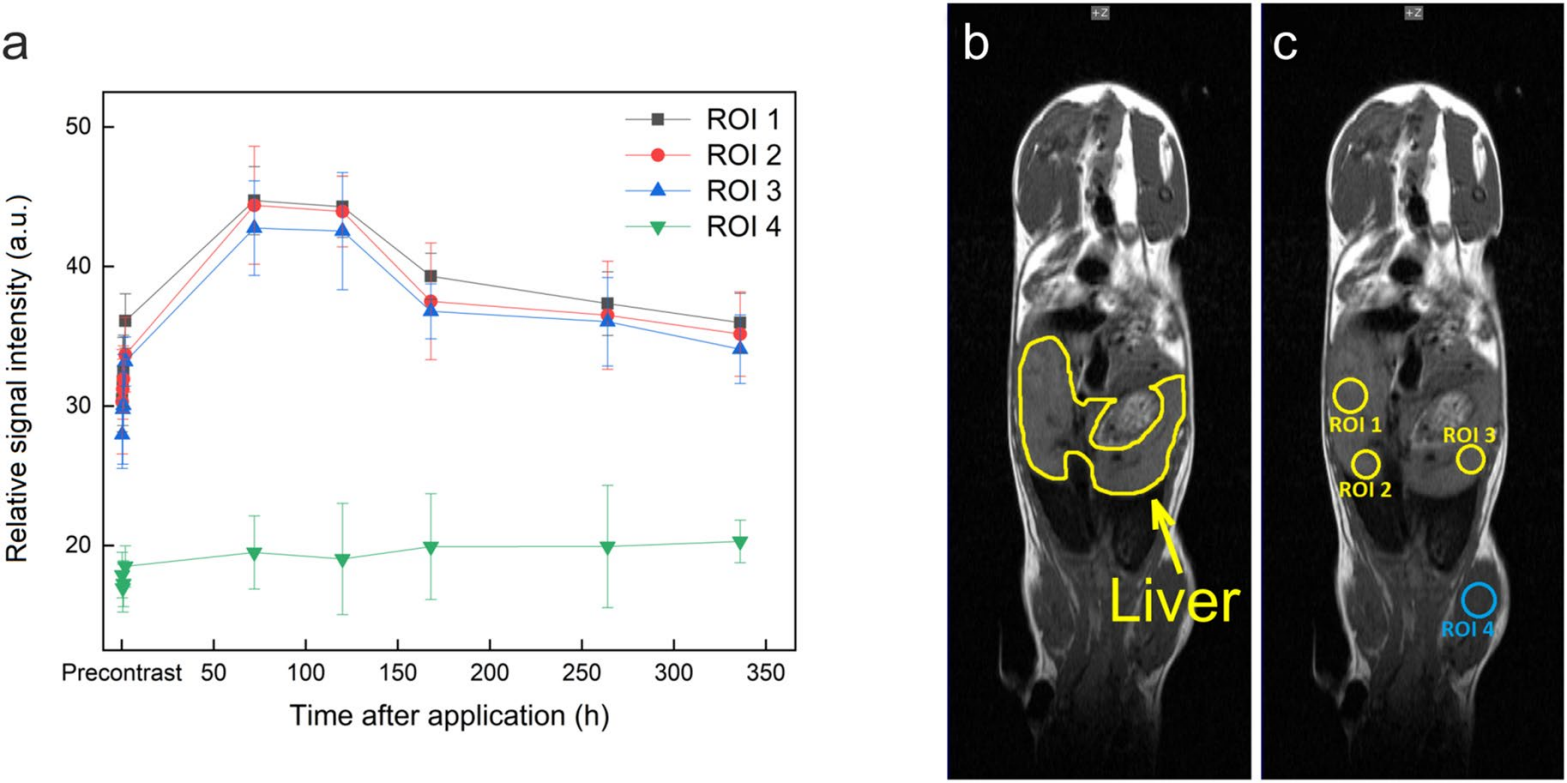
(**a**) MRI relative signal intensity of targeted tissues in regions of interest (ROIs) in liver (ROI 1–ROI 3) and muscle (ROI 4) before and after the intravenous application of NaGdF_4_:Yb^3+^/Er^3+^@PEG nanoparticles. The results show the mean value ± standard deviation, which were evaluated from *T*_1_-weighted images in selected liver ROIs and related to the reference signal (ROI 4 in muscle). (**b**) Liver position marked in mouse body on respective image. (**c**) Regions of interest (ROIs) were chosen in three different parts of the liver (ROI 1–3) and compared with a reference ROI 4 (muscle in the left or right limb). Both liver and ROI images were acquired from precontrast scans.

**Figure 7:**
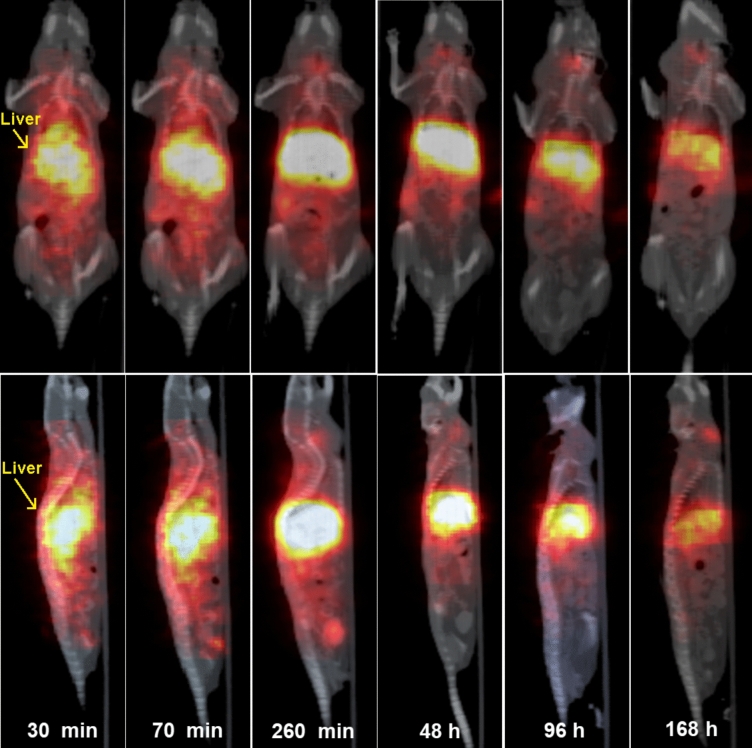
In vivo SPECT/CT images of a representative mouse at various time points after the intravascular application of NaGdF_4_:Yb^3+^/Er^3+^@PEG-^125^I nanoparticles; both coronal and sagittal orientation.

Biodistribution of the particles was investigated in mice; the particles were evidently nontoxic, as animal’s feed habits were unchanged after the particle administration, the mice were active and interacted with each other during the study. The weight of animals did not significantly change, nor altered posture and/or fur appearance (typical signs of discomfort or toxicity of the injected compound) has been observed. The in vivo MRI results revealed an intense signal in the liver caused by the accumulation of the NaGdF_4_:Yb^3+^/Er^3+^@PEG particles (Fig. 5). Comparing the reference region of interest (ROI; shoulder muscle) versus three different ROI in the liver revealed a maximum signal increase at 72 h after particle application (Fig. 6). Additionally, SPECT/CT in vivo measurements demonstrated the fast accumulation of NaGdF_4_:Yb^3+^/Er^3+^@PEG-^125^I nanoparticles in the liver. The particles circulated for at least 4 h after the application. The activity remained in the liver and the signal peaked after 48 h. The signal then started to decline due to detoxication of the particles via the hepatobiliary route (Fig. 7) that is a typical excretion way from the liver. A weak signal was also observed in the thyroid gland, which was caused by free ^125^I leaking from the iodinated shell of the particles. The SPECT measurement proved to be more sensitive for determining the particle biodistribution than MRI. The MRI results reported only the peak values at longer times. The initial accumulation that was observable on the SPECT image within 2 h was not seen on the MRI image. Nevertheless, MRI provides indispensable information about the exact position of the nanoparticles within the soft tissues.

## Discussion

High-temperature coprecipitation of lanthanide chlorides produced NaGdF_4_:Yb^3+^/Er^3+^ and NaGdF_4_:Yb^3+^/Tm^3+^ UCNPs. Compared to other conventional synthetic procedures, this approach brings several benefits regarding the precise control of the crystal structure, morphology, and uniformity of the resulting nanoparticles. The particle size distribution was quite narrow and more uniform compared to that in other recent studies^56,57^. The particle uniformity is a key criterium for the subsequent application of the particles in biomedicine^18^. The thoroughly washed nanoparticles could not be directly transferred in a phosphate buffer, which is required for biomedical applications, because they immediately aggregated and formed a turbid colloidal solution (Fig. S2b). Therefore, the particles were modified with PEG-Ner to provide them with very good colloidal stability not only in water (Fig. S2c) but also in PBS (Fig. S2d). This stability was higher than that of cancer cell membrane-coated NaGdF_4_:Yb,Tm@NaGdF_4_ particles, which were stable in PBS for only 3 days^58^. Additional advantage of phosphonate-functionalized NaGdF_4_:Yb/Er UCNPs consists in their enhanced accumulation in bones, which could be helpful for treatment and imaging of bone diseases^59^. Upconversion photoluminescence measurements of the NaGdF_4_:Yb^3+^/Er^3+^ and NaGdF_4_:Yb^3+^/Tm^3+^ nanoparticles confirmed the characteristic strong green/red (520, 540, and 650 nm) and UV/blue/NIR emissions (447, 473, and 800 nm). The NaGdF_4_:Yb^3+^/Er^3+^@PEG single-particle investigation revealed that the high luminescence intensity was not compromised by the surface modification. The luminescence intensity map of tens of individual PEG-functionalized upconversion nanoparticles monitored by the widefield microscopy made statistical analysis of their optical properties possible. The single-particle studies indicated that the surface engineering did not affect the luminescence intensity of the NaGdF_4_:Yb^3+^/Er^3+^ nanoparticle, which confirmed that their high homogeneity was due to the equal width of the luminescence intensity distribution. Single particle imaging is thus a powerful tool that has huge potential in bioimaging and tracing of specific proteins in theranostic applications. Considering in vivo upconversion imaging, it is much more challenging compared to in vitro studies especially due to relatively low emission wavelengths of UCNPs leading to significant absorption of emitted light in the tissues. The efficient optical imaging of deeper tissues needs excitation and emission wavelengths that fit into the “NIR imaging window”^60^. Our NaGdF_4_:Yb^3+^/Tm^3+^ particles show strong emission at 800 nm, which is the optimal wavelength for imaging with low hemoglobin and water absorption. The particles are thus a potential candidate also for in vivo optical imaging.

The NaGdF_4_:Yb^3+^/Er^3+^@PEG nanoparticles were found to be biocompatible, not significantly affecting viability of the HeLa or HF cells at concentrations < 400 μg/ml. At 400 μg/ml, the HF cells were slightly more sensitive than the HeLa cells. The difference in the behavior of the HeLa and HF cells was attributed to their different metabolism. HF cells are primary fibroblasts that are more sensitive to incubation with potentially toxic agents than the tumor HeLa cells, which are more adaptable to various anticancer drugs, for instance. Similar results, where noncancer cell lines, especially HF cells, showed different metabolic activity compared to cancer cell lines^61^ or higher sensitivity to treatment with various nanomaterials, were demonstrated also in other studies^35,62^.

MRI and relaxometric studies suggested that the particles possessed suitable paramagnetic properties that will allow for the application of the NaGdF_4_:Yb^3+^/Er^3+^@PEG particles as a novel contrast probe for MRI; this is in agreement with an earlier published report on sodium gluconate-coated lanthanide-doped upconversion nanoparticles^63^. Moreover, we decided to add the third imaging modality to the NaGdF_4_:Yb^3+^/Er^3+^@PEG particles by their radiolabeling that is a rarely used modification of UCNPs^64,65^. This approach enabled us to use SPECT method, rather sparsely exploited technique for the determination of particle biodistribution, which allows higher spatial resolution and more reliable and accurate nanoparticle quantification in deeply located organs than fluorescence employed in most current studies^56,57,66^. Pilot in vivo MRI/SPECT multimodal imaging of the NaGdF_4_:Yb^3+^/Er^3+^@PEG nanoparticles proved that they circulated in the blood stream of an experimental animal for at least 4 h, though rapid uptake to the liver was observed with a maximum signal 2–3 days after intravenous application. The blood circulation time was prolonged compared to that achieved with poly(ethylene glycol) diglycidyl ether-crosslinked poly(maleic anhydride-*alt*-1-octadecene)-coated NaYF_4_:Yb^3+^Tm^3+^@NaYF_4_ nanoparticles that circulated for up to 1 h^67^.

Our multimodal probes are constructed to allow SPECT imaging with ^125^I. SPECT is more available and widely used and cheaper than PET in systems for NIR/MRI/PET applications reported earlier^68^. ^125^I-labelled SPECT probes have also advantage of considerably lower energy than PET probes (35 keV *vs*. 511 keV) and relatively long half-time allowing to use them in longitudinal studies that cannot be performed with short-living high-energy PET isotopes. Moreover, PET intrinsic resolution cannot go under positron range 1–3 mm^69^, while ultra-SPECT resolution can be significantly better^70^. The multimodal character of the NaGdF_4_:Yb^3+^/Er^3+^@PEG nanoparticles allows to use them in laboratories equipped with any of the imaging methods (optical imaging, SPECT, MRI). Applying two or more of these methods to the imaged object allows very precise characterization of the diseased tissue.

## Conclusion

Ultimately, the NaGdF_4_:Yb^3+^/Er^3+^@PEG nanoprobes were not only luminescent, but also enabling MR imaging. Depending on the selection of the dopant ions, the size of the particles was controlled in the range of 13–18 nm with a relatively narrow particle size distribution. The particles proved to be biocompatible, dispersible, and stable in phosphate buffer media. The colloidal stability of the NaGdF_4_:Yb^3+^/Er^3+^ particles in PBS was achieved by a unique, rapid, and facile surface modification with PEG-neridronate, which amounted to 20 wt.% according to TGA; this is in agreement with our previous report on the NaYF_4_:Yb^3+^/Er^3+^@PEG nanoparticles^35^. The particles exhibited NIR-to-UV/Vis photoluminescence, sufficient relaxivity enabled contrast in MRI, ^125^I labeling allowed SPECT imaging, and thus their biodistribution and fate could be monitored in vivo. Prolonged blood circulation time, targeting specific tissues or cell labeling applications would require additional functionalization of the particles. Multimodality of NIR luminescence/MRI/SPECT/CT successfully applied in one system can potentially reduce overall cost and combine advantages of the three individual imaging techniques. The recent availability of a new generation of optical imagers allowing imaging in NIR I and NIR II makes the newly developed NaGdF_4_:Yb^3+^/Er^3+^@PEG nanoparticles perspective for use in NIR bioimaging and theranostics. This makes the particles a prospective diagnostic and/or theranostic platform for the multimodal imaging of serious disorders.

## Supplementary information

Supplementary information.
