# Supplementary material for: Highly colloidally stable trimodal 125I-radiolabeled PEG-neridronate-coated upconversion/magnetic bioimaging nanoprobes
Source: Sci Rep. 2020 Nov 18;10:20016. doi: 10.1038/s41598-020-77112-z (PMC7675969; doi:10.1038/s41598-020-77112-z)
Supplement: Supplementary file 1 — Supplementary information. [file 41598_2020_77112_MOESM1_ESM.docx]

**ELECTRONIC SUPPLEMENTARY INFORMATION**

**Highly colloidally stable three-modal ^125^I-radiolabeled PEG-neridronate-coated upconversion/magnetic bioimaging nanoprobes**

Uliana Kostiv, Jan Kučka, Volodymyr Lobaz, Nikolay Kotov, Olga Janoušková, Miroslav Šlouf, Bartosz Krajnik, Artur Podhorodecki, Pavla Francová, Luděk Šefc, Daniel Jirák, Daniel Horák

**Table S1.** *T*_1_ and *T*_2_ relaxation times and *R_1_* and *R_2_* relaxation rates of NaGdF_4_:Yb^3+^/Er^3+^ nanoparticles at different concentrations. Measured at 37 °C and 1.41 T.

| Concentration (mM) | *T*_1_ (ms) | *R*_1_ (s^-1^) | *T*_2_ (ms) | *R*_2_ (s^-1^) |
| --- | --- | --- | --- | --- |
| 0 | 3,726.0±103.1 | 0.3 | 3,440.0±5.6 | 0.3 |
| 0.55 | 1,990.0 ±49.7 | 0.5 | 420.9±2.1 | 2.4 |
| 1.09 | 1,546.7±36.8 | 0.6 | 237.9±1.9 | 4.2 |
| 2. 73 | 900.0±16.3 | 1.1 | 40.3±1.1 | 24.8 |
| 5.5 | 310.8±7.3 | 3.2 | 21.5±0.7 | 46.5 |
| 10.9 | 163.9±2.1 | 6.1 | 10.4±0.5 | 96.2 |

**Table S2.** *T*_1_ and *T*_2_ relaxation times and *R_1_* and *R_2_* relaxation rates of NaGdF_4_:Yb^3+^/Er^3+^@PEG nanoparticles at different concentrations. Measured at 37 °C and 1.41 T.

| Concentration (mM) | *T*_1_ (ms) | *R*_1_ (s^-1^) | *T*_2_ (ms) | *R*_2_ (s^-1^) |
| --- | --- | --- | --- | --- |
| 0 | 3,726.0±103.1 | 0.3 | 3,440.0±5.6 | 0.3 |
| 0.54 | 1,351.7±15.5 | 0.7 | 225.9±11.6 | 4.4 |
| 1.07 | 809.7±9.3 | 1.2 | 111.3±5.4 | 9.0 |
| 2.68 | 362.6±0.6 | 2.8 | 43.9±2.0 | 22.8 |
| 5.35 | 183.5±0.6 | 5.5 | 22.1±1.0 | 45.3 |
| 10.7 | 95.2±0.8 | 10.5 | 11.3±0.6 | 88.5 |

**Figures**


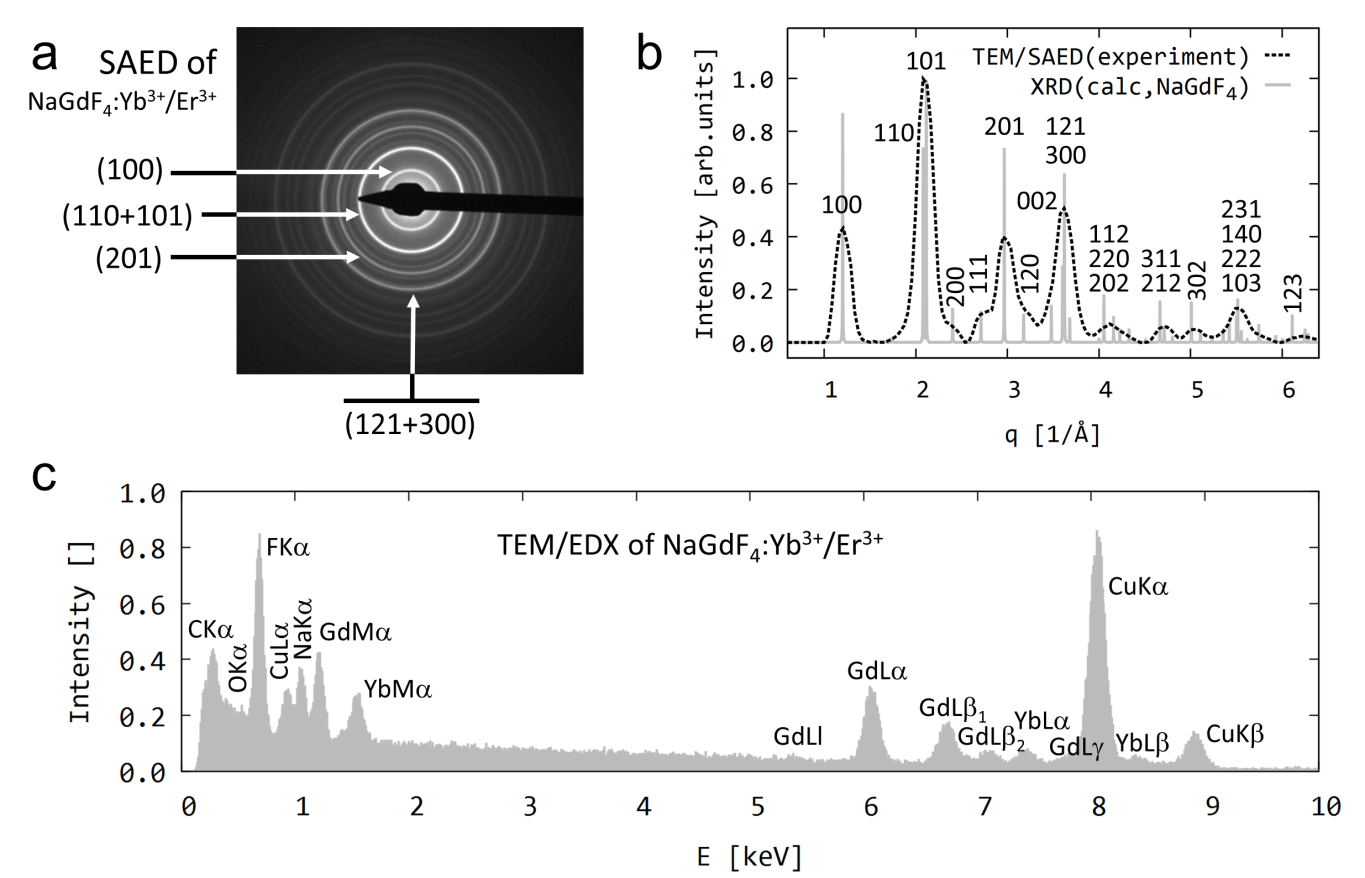


**Figure S1**. TEM analysis of the crystal structure and the elemental composition of the NaGdF_4_:Yb^3+^/Er^3+^ nanoparticles. (a) Selected area electron diffraction (SAED) pattern, (b) comparison of the experimental SAED pattern with theoretically calculated X-ray diffraction (XRD) pattern corresponding to the hexagonal phase of NaGdF_4_, and (c) energy-dispersive (EDX) spectrum.

**
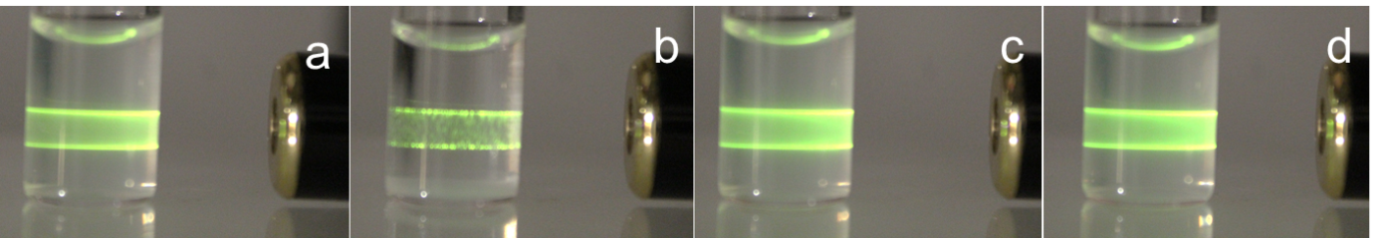
**

**Figure S2.** Images of upconversion emission of (a, b) NaGdF_4_:Yb^3+^/Er^3+^ and (c, d) NaGdF_4_:Yb^3+^/Er^3+^@PEG nanoparticles in (a, c) water and (b, d) 0.01 M PBS buffer. Excitation at 980 nm, laser with a power of 400 mW.

**
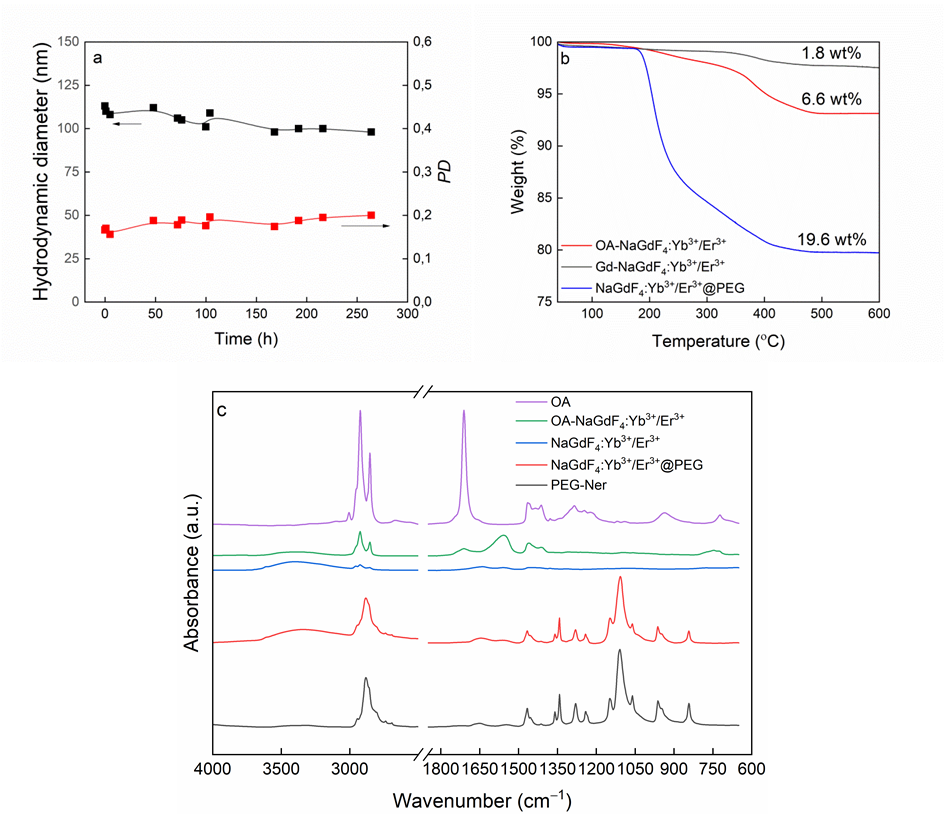
Figure S3**. (a) Hydrodynamic diameter *D*_h_ and polydispersity *PD* of NaGdF_4_:Yb^3+^/Er^3+^@PEG nanoparticles in PBS, (b) TGA thermograms, and (c) ATR FTIR spectra of NaGdF_4_:Yb^3+^/Er^3+^ and NaGdF_4_:Yb^3+^/Er^3+^@PEG nanoparticles.


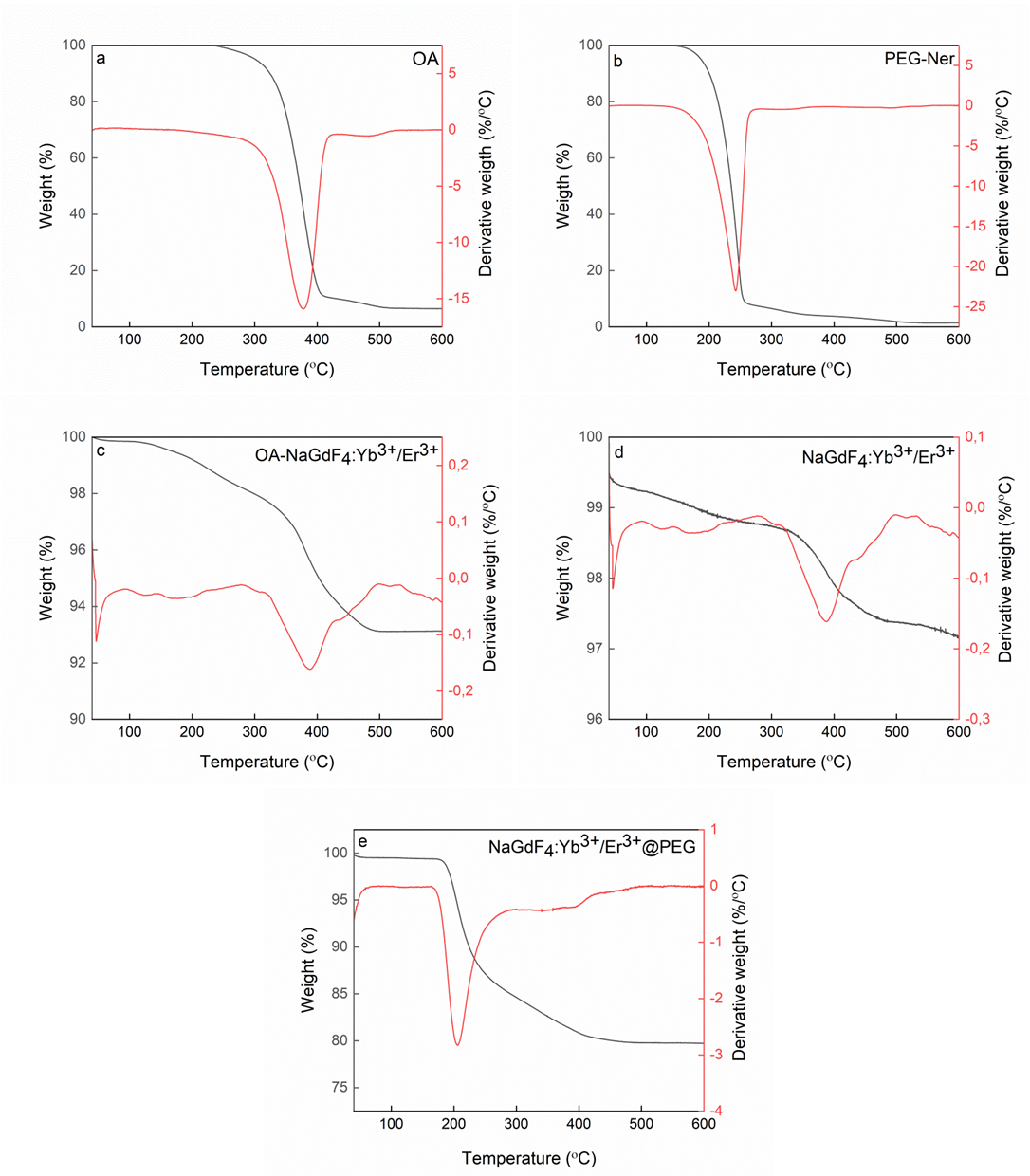


**Figure S4.** TGA and the first derivative of TGA curve of (a) oleic acid (OA), (b) PEG-Ner, (c) OA-NaGdF_4_:Yb^3+^/Er^3+^, (d) NaGdF_4_:Yb^3+^/Er^3+^, and (e) NaGdF_4_:Yb^3+^/Er^3+^@PEG nanoparticles.


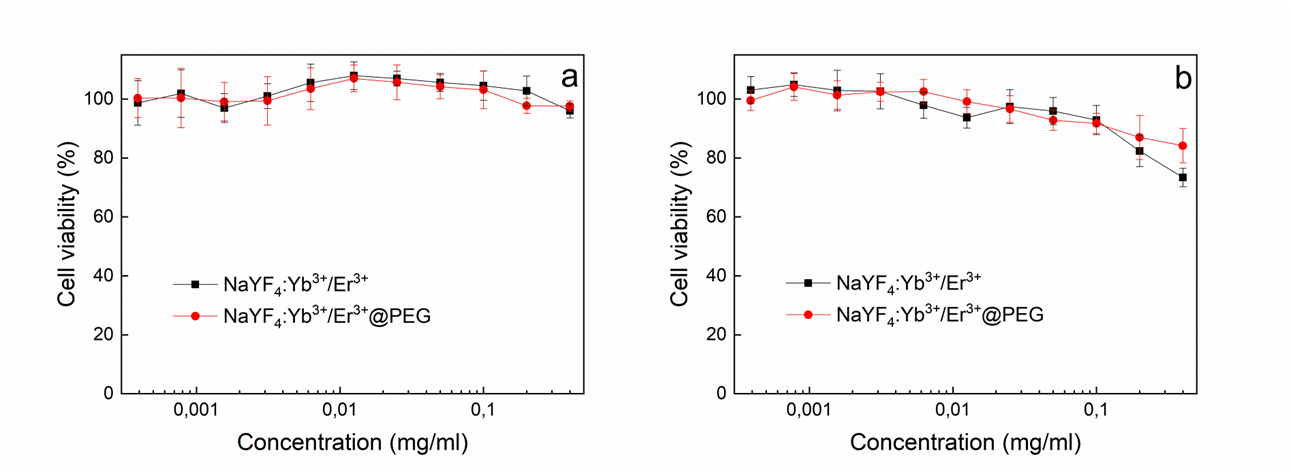


**Figure S5.** Viability of (a) HeLa and (b) HF cells treated with different concentrations of NaGdF_4_:Yb^3+^/Er^3+^ and NaGdF_4_:Yb^3+^/Er^3+^@PEG nanoparticles.

|  |  |
| --- | --- |
|  |  |

**Figure S6.** Dependence of (a, c) *R*_1_ and (b, d) *R*_2_ relaxation rates on the concentration of (a, b) NaGdF_4_:Yb^3+^/Er^3+^ and (c, d) NaGdF_4_:Yb^3+^/Er^3+^@PEG nanoparticles measured at 37 °C and 1.41 T.
